# Fed-batch enzymatic hydrolysis of alkaline organosolv-pretreated corn stover facilitating high concentrations and yields of fermentable sugars for microbial lipid production

**DOI:** 10.1186/s13068-019-1639-9

**Published:** 2020-01-22

**Authors:** Zhiwei Gong, Xuemin Wang, Wei Yuan, Yanan Wang, Wenting Zhou, Guanghui Wang, Yi Liu

**Affiliations:** 1 School of Chemistry and Chemical Engineering, Wuhan University of Science and Technology, 947 Heping Road, Wuhan, 430081 People’s Republic of China; 2 State Key Laboratory Breeding Base of Dao-di Herbs, National Resource Center for Chinese Materia Medica, China Academy of Chinese Medical Sciences, Beijing, 100700 People’s Republic of China; 3 HuBei Province Key Laboratory of Coal Conversion and New Carbon Materials, Wuhan University of Science and Technology, Wuhan, 430081 People’s Republic of China; 4 College of Chemistry and Molecular Sciences, Wuhan University, Wuhan, 430072 People’s Republic of China

**Keywords:** Corn stover, Alkaline organosolv pretreatment, High solids loading, Fed-batch enzymatic hydrolysis, Fermentable sugars, Lipids

## Abstract

**Background:**

Lignocellulosic biomass has been commonly regarded as a potential feedstock for the production of biofuels and biochemicals. High sugar yields and the complete bioconversion of all the lignocellulosic sugars into valuable products are attractive for the utilization of lignocelluloses. It is essential to pretreat and hydrolyze lignocelluloses at high solids loadings during industrial processes, which is more economical and environmentally friendly as capital cost, energy consumption, and water usage can be reduced. However, oligosaccharides are inevitably released during the high solids loading enzymatic hydrolysis and they are more recalcitrant than monosaccharides for microorganisms.

**Results:**

A fed-batch enzymatic hydrolysis of corn stover pretreated by the sodium hydroxide–methanol solution (SMs) at high solids loading was demonstrated to reach the high concentrations and yields of fermentable sugars. Glucose, xylose, cello-oligosaccharides, and xylo-oligosaccharides achieved 146.7 g/L, 58.7 g/L, 15.6 g/L, and 24.7 g/L, respectively, when the fed-batch hydrolysis was started at 12% (w/v) solids loading, and 7% fresh substrate and a standardized blend of cellulase, β-glucosidase, and hemicellulase were fed consecutively at 3, 6, 24, and 48 h to achieve a final solids loading of 40% (w/v). The total conversion of glucan and xylan reached 89.5% and 88.5%, respectively, when the oligosaccharides were taken into account. Then, a fed-batch culture on the hydrolysates was investigated for lipid production by *Cutaneotrichosporon oleaginosum*. Biomass, lipid content, and lipid yield were 50.7 g/L, 61.7%, and 0.18 g/g, respectively. The overall consumptions of cello-oligosaccharides and xylo-oligosaccharides reached 74.1% and 68.2%, respectively.

**Conclusions:**

High sugars concentrations and yields were achieved when the enzyme blend was supplemented simultaneously with the substrate at each time point of feeding during the fed-batch enzymatic hydrolysis. Oligosaccharides were co-utilized with monosaccharides during the fed-batch culture of *C. oleaginosum*. These results provide a promising strategy to hydrolyze alkaline organosolv-pretreated corn stover into fermentable sugars with high concentrations and yields for microbial lipid production.

## Background

Lignocellulosic biomass has already been widely regarded as a potential feedstock for the production of biofuels and biochemicals due to its low cost, abundance, and sustainability [1, 2]. A variety of fermentable sugars including glucose, xylose, arabinose, mannose, galactose, cellobiose, etc., can be released from lignocelluloses. Enzymatic hydrolysis has been predominantly applied for the production of fermentable sugars from lignocelluloses for its high selectivity and mild processing conditions [3]. However, the recalcitrance of biomass impedes degradation of lignocelluloses [1, 4]. Over the past decades, various pretreatment techniques have been developed to address biomass recalcitrance by disrupting physical barriers of cell wall, reducing cellulose crystallinity, and removing lignin and hemicellulose, for the purpose of facilitating the enzymatic hydrolysis of lignocelluloses into fermentable sugars [5–7]. The numerous pretreatment approaches can be divided into four major categories, including physical, chemical, physicochemical, and biological ones [8]. Among them, alkali pretreatment has attracted extensive attentions, for its capability of preserving a large proportion of cellulose and removing lignin under mild processing conditions [9, 10].

Low solids loadings are generally applied for enzymatic hydrolysis to ensure an adequate mixing between cellulase and cellulosic materials [11]. Nevertheless, low solids loadings generate low concentrations of sugars, thus resulting in low products titers and high costs of separation and purification [12, 13]. It is essential to pretreat and hydrolyze lignocelluloses at high solids loadings during industrial processes, which is more economical and environmentally friendly by reducing capital cost, energy input, and water usage [14–17]. However, when the enzymatic hydrolysis is directly conducted at very high solids loadings, the mixing is difficult to perform when mixing strategies are not applied, which exerts negative effects on the mass and heat transfer efficiency while severely hindering the hydrolysis process [16, 18]. To avoid these adverse effects and maximize release of sugars, fed-batch strategy has been proposed [19, 20]. For a typical enzymatic fed-batch hydrolysis, the hydrolysis is initiated at a relatively low solids loading, commonly less than 15% (w/v). Substrate is then successively fed to maintain the solids content at a relative low level, and finally, the high solids loadings are achieved.

Oligosaccharides are released along with monosaccharides during enzymatic hydrolysis of lignocelluloses at high solids loadings [21, 22]. For example, oligosaccharides accounted for approximately 21.8% of the total soluble sugars in the ammonia fiber explosion (AFEX)-pretreated corn stover enzymatic hydrolysates [21]. Unfortunately, many microorganisms lack the ability to metabolize oligosaccharides. The non-consumed oligosaccharides will be discharged along with fermentation wastewater, which means the loss of sugars. For example, the accumulation of oligosaccharides represents a yield loss as these sugars are recalcitrant for most industrial ethanol-producing microorganisms [22]. Moreover, oligosaccharides have been identified as strong inhibitors of cellulase [22, 23]. The addition of xylanase and β-xylosidase has been proposed to address the inhibition of cellulase by xylo-oligosaccharides [24]. Complete enzymatic hydrolysis of the sugar polymers in cellulosic materials into monosaccharide has always been the routine task of the researchers, which requires the synergy of many hydrolytic enzymes with optimized composition. However, it requires a high enzyme cost severely impeding the commercial success. Various pretreatment strategies have been developed to remove lignin and hemicellulose in order to obtain a high cellulose content of the regenerated biomass, facilitating a glucose-rich hydrolysate [25–28]. It is worth noting that xylose and oligosaccharides are capable to be metabolized for biofuels and biochemicals production by a few wild-type and engineering microorganisms [29–32]. Thus, it is not a necessity to prepare glucose-rich hydrolysates. As reported by Yang and his colleagues, the hydrolysis yield was strongly inhibited by glucose in excess of 100 g/L [28]. Generation of suitable proportions of xylose and oligosaccharides may be effective in mitigating the strong inhibitory effects of high-concentration glucose and facilitating fermentable sugars with high concentrations and yields.

The traditional alkali pretreatment suffers from some issues such as high energy consumption, strong corrosion to the reactor, low solids loadings, and wastewater discharge. Alkaline organosolv pretreatment is promising for the preservation of both cellulose and hemicelluloses [33]. In the present study, corn stover was effectively pretreated by sodium hydroxide–methanol solution (SMs) at high solids loading of 50% (w/v). This pretreatment has some advantages over the traditional alkali pretreatment using water as solvent, namely significantly lowering the energy consumption due to a higher solids loading and a lower specific heat capacity of methanol and easily achieving high solids by removal of methanol to facilitate the high solids enzymatic hydrolysis. To obtain high concentrations and yields of fermentable sugars from the SMs-pretreated corn stover, the fed-batch enzymatic hydrolysis was adopted and a variety of feeding strategies were investigated. Co-fermentation of monosaccharides and oligosaccharides within the hydrolysate by the oleaginous yeast *C. oleaginosum* using a fed-batch culture mode was then investigated to reach high lipid content and yield. This work should offer valuable information for obtaining high concentrations and yields of fermentable sugars for microbial lipid production.

## Results and discussion

### The compositional changes of corn stover after the alkaline organosolv pretreatment at high solids loading

The compositions of the raw and the SMs-pretreated corn stover are shown in Table 1. The pretreated corn stover contained 46.5% glucan, 25.8% xylan, 8.6% lignin, and 3.2% ash (Table 1, Entry 2). The recovery of the glucan and xylan was 97.4% and 89.4%, respectively, suggesting that a large proportion of the sugar polymers remained in the solid phase. Interestingly, over 70% of lignin was removed by the SMs pretreatment, which was consistent with the traditional alkaline aqueous solution pretreatment that could dissolve lignin considerably [34].

**Table 1:** Chemical compositional results of the raw and the SMs-pretreated corn stover

Entry	Sample	Solid recovery (%, w/w)	Composition (%, w/w)			
			Glucan	Xylan	Total lignin	Ash
1	Raw corn stover	–	35.9 (± 0.3)	21.7 (± 0.5)	22.1 (± 0.3)	2.3 (± 0.2)
2	Pretreated corn stover	75.2 (± 0.5)	46.5 (± 0.4)	25.8 (± 0.4)	8.6 (± 0.1)	3.2 (± 0.3)

### Effects of solids loadings on the release of sugars during batch enzymatic hydrolysis

**Fig. 1:**
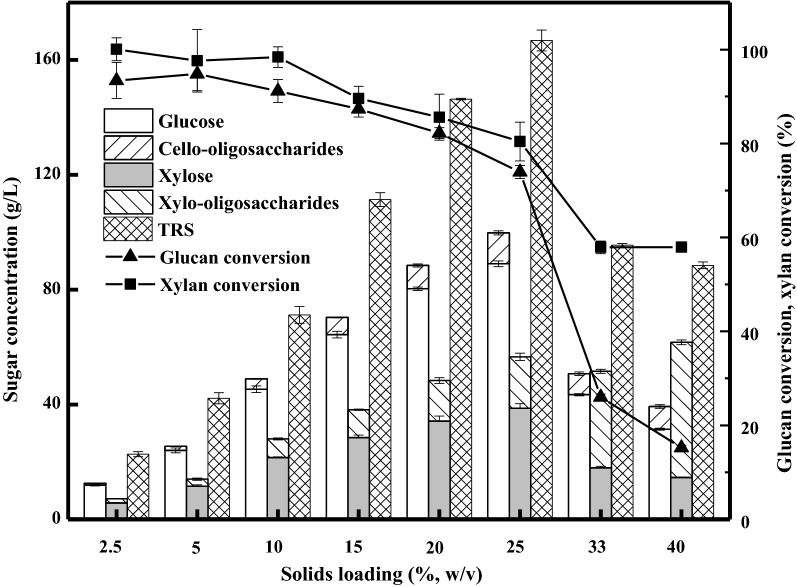
Sugar concentrations and conversions for the batch enzymatic hydrolysis at solids loadings ranging from 2.5 to 40% (w/v). The enzyme blend was loaded at 15 mg protein/g glucan. The pretreated corn stover was hydrolyzed at 50 °C, pH 4.8, and 200 rpm for 72 h

The solids loading exerts significant effects on the saccharification efficiency and yield. Herein, batch enzymatic hydrolysis of the pretreated corn stover at solids loadings varying from 2.5 to 40% (w/v) was investigated and compared. The hydrolysis was conducted at 50 °C for 72 h. As shown in Fig. 1, glucose and xylose were 12.0 g/L and 5.7 g/L when the solids loading was 2.5%, corresponding to the yields of 89.6% and 77.4%, respectively. In addition, 0.6 g/L cello-oligosaccharides and 1.5 g/L xylo-oligosaccharides were observed in the hydrolysates. Surprisingly, the total conversions of glucan and xylan reached 93.5% and 100.1%, respectively, when the oligosaccharides were taken into account. The total reducing sugars (TRS) released from corn stover increased linearly with a highly significant correlation (y = 6.94x + 5.45, R^2^ = 0.9954), when the solids loading increased from 2.5 to 15%. The carbohydrate polymers within lignocelluloses could be well depolymerized into fermentable sugars by the commercial enzyme cocktail, when the hydrolysis was performed at relatively low solids loadings. Glucose and xylose increased on a continued basis, while their yields demonstrated the opposite trends as the solids loading rose from 15 to 25%. Glucose, xylose, and TRS were in sharp decline, when the solids loadings rose to 33% and 40%. Only 31.4 g/L glucose and 14.6 g/L xylose were observed in the hydrolysates at 40% solids loading, corresponding to the theoretical yields of 11.9% and 12.3%, respectively. Cello-oligosaccharides and xylo-oligosaccharides reached 8.0 g/L and 49.0 g/L, causing the glucan and xylan conversions of 15.3% and 57.9%, respectively. Direct enzymatic hydrolysis at high solids loading led to an increased viscosity and a reduced mass transfer efficiency, which resulted in the low concentrations and yields of fermentable sugars [16, 18, 35]. In fact, there was no visible liquid spotted during enzymatic hydrolysis at 40% solids loading, which was not conducive to the interaction between the enzymes and the substrate.

The vast majority of industrial fermentation processes are proposed using fed-batch culture mode [36, 37]. The high concentration of fermentable sugars is a necessity for the subsequent feeding process over the course of fed-batch fermentation. Enzymatic hydrolysis at high solids loadings is feasible for deriving high-concentration sugars while reducing water usage, capital investment, and energy consumption significantly. Nevertheless, batch enzymatic hydrolysis at very high solids directly suffers from the difficulty in mixing and low mass and heat transfer efficiency, which results in low hydrolysis yields.

### Optimization of time point of feeding during the fed-batch enzymatic hydrolysis

**Fig. 2:**
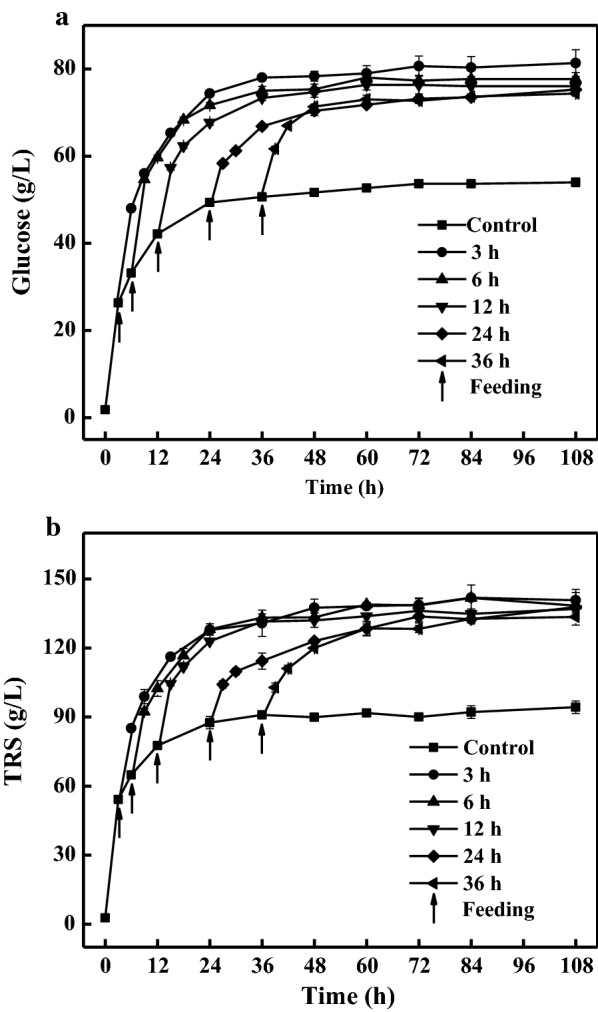
Effects of different time points of feeding on the release of glucose (**a**) and TRS (**b**). The enzymatic hydrolysis was initiated at 12% (w/v) solids loading, and 7% solids were separately fed at different time points to reach the final solids loading of 19% (w/v)

It is challenging to reach very high concentrations and yields of fermentable sugars by one-step feeding without mixing strategies. To resolve the mass transfer issues under high solids loadings, the batch enzymatic hydrolysis with mechanical stirring has been performed at solids loadings of 28% (w/w) and 30% (w/w), respectively [12, 38]. In addition, fed-batch hydrolysis mode has been applied to achieve high solids loadings [16, 28]. In this study, a fed-batch enzymatic hydrolysis strategy was attempted to achieve this goal. When the enzymatic hydrolysis was conducted at an initial solids loading exceeding 15% (w/v), the mixing became difficult, which was adverse to the pH adjustment and mass transfer. The optimal initial solids loading was 12% (w/v) (Additional file 1), which was in accordance with the previous published results [16, 28]. The time point of feeding represents a crucial factor in fed-batch hydrolysis process. In this study, the hydrolysis was initiated at 12% (w/v) solids loading and the pretreated corn stover (7%, w/v, based on the final solids loading) was separately fed after 3, 6, 12, 24, or 36 h, respectively, to reach the final solids loading of 19% (w/v). Glucose and TRS were measured over the course of hydrolysis. As illustrated in Fig. 2, the earlier the time point of feeding was, the faster the hydrolysis reached the platform stage (Fig. 2). Finally, 81.3 g/L of glucose and 140.8 g/L of TRS were observed in the hydrolysates when the corn stover was fed at the time point of 3 h. Glucose and TRS were reduced constantly when the time point of feeding extended from 3 h to 24 h. It should be noted that there were no significant (P > 0.05) negative effects on the hydrolysis when the feeding intervals were limited to within 12 h. The maximal concentrations of glucose and TRS decreased by 8.6% and 5.2%, respectively, when the feeding time was extended to 36 h, which was largely attributed to a shorter time of interaction between the enzymes and the feeding substrate. In addition, part of the enzymes might be inactivated as the hydrolysis proceeded. Thus, it was beneficial to feed earlier during the fed-batch enzymatic hydrolysis without impeding the mass transfer, which was crucial for enhancing the hydrolysis efficiency and shortening the total hydrolysis time. Similarly, the substrates have been proposed to be fed early to reduce the hydrolysis time during the fed-batch process [19, 28].

### Fed-batch enzymatic hydrolysis at various solids loadings

**Fig. 3:**
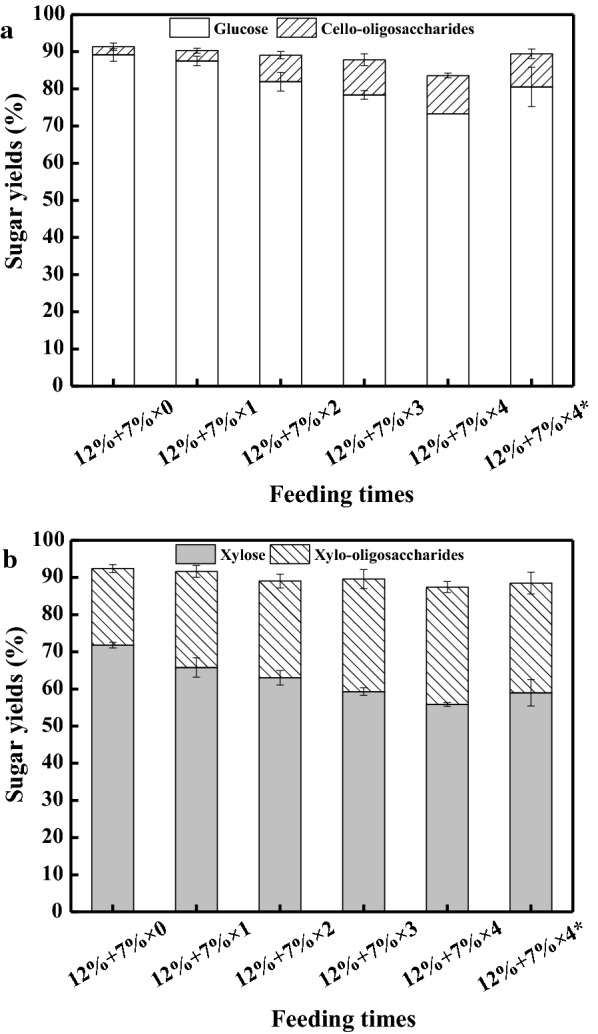
Sugars yields of the fed-batch enzymatic hydrolysis at solids loadings varying from 12 to 40% (w/v). **a** yields of glucose and cello-oligosaccharides. **b** yields of xylose and xylo-oligosaccharides. *The enzyme blend was fed four times simultaneously with the substrate

The fed-batch hydrolysis was initiated at 12% (w/v) solids loading, and 7% (w/v) pretreated corn stover was fed once, twice, three times, and four times, respectively. The results are presented in Table 2 and Fig. 3. Glucose and xylose reached 54.0 g/L and 23.8 g/L, corresponding to the theoretical yields of 89.2% and 71.8%, respectively, when enzymatic hydrolysis was conducted at 12% solids loading (Table 2, Entry 1). The total conversions of glucan and xylan reached 91.3% and 92.4%, respectively, when the oligosaccharides were taken into account (Fig. 3). It was indicated that the SMs pretreatment was promising to destroy the recalcitrant structure of corn stover, thus facilitating the degradation of cellulose and hemicellulose into monosaccharides and oligosaccharides. With the increase in feeding times, the concentrations of monosaccharides and oligosaccharides were gradually increased, while the monosaccharides yields decreased on a continued basis. When corn stover was fed once, the total conversions of glucan and xylan reached 90.3% and 91.6% (Fig. 3), slightly lower than those from the batch hydrolysis without significant variances (P > 0.05). When corn stover was fed twice, glucose and xylose increased dramatically to 102.0 g/L and 42.9 g/L, while their yields decreased to 81.9% and 63.0%, respectively (Table 2, Entry 3). The total conversions of glucan and xylan slightly decreased to 89.1% and 89.0%, respectively, without significant variances (P > 0.05) (Fig. 3). When the corn stover was fed three times to the final solids loading of 33%, glucose and xylose yields fell to 78.4% and 59.3%, respectively (Table 2, Entry 4). The total conversions of glucan and xylan were 87.8% and 89.6%, respectively (Fig. 3). When the substrate (7%) was fed four times to reach a final solids loading of 40%, the yields of glucose and xylose decreased to 73.3% and 55.8%, respectively, which were significantly (P < 0.05) lower than those from the batch hydrolysis. Cello-oligosaccharides and xylo-oligosaccharides reached 17.9 g/L and 27.2 g/L as a maximum, respectively. The total conversions of glucan and xylan decreased to 83.6% and 87.4%, respectively (Fig. 3). Cello-oligosaccharides and xylo-oligosaccharides have been identified as strong inhibitors of cellulase [22–24, 39]. Here, the accumulation of oligosaccharides probably contributed to the significant decline in the monosaccharides yields. The time course of sugar evolutions during the fed-batch hydrolysis demonstrated that most of the sugars were released within 72 h even if the substrate was fed four times (Additional file 2).

**Table 2:** Results of fed-batch enzymatic hydrolysis with different feeding times and the chemical compositions of the solid residues after enzymatic hydrolysis

Entry	Solids loading (%, w/v)	Feeding times (time point of feeding, h)^b^	Hydrolysates					Solid residues after enzymatic hydrolysis			
			Sugar concentration (g/L)					Composition (%, w/w)			
			Glucose	Cello-oligosaccharides	Xylose	Xylo-oligosaccharides	TRS	Glucan	Xylan	Total lignin	Ash
1	12	–	54.0 (± 1.0)	1.7 (± 0.5)	23.8 (± 0.3)	6.0 (± 0.3)	94.3 (± 2.7)	14.4 (± 0.3)	7.8 (± 0.4)	37.6 (± 1.0)	29.5 (± 0.2)
2	19	1 (3)	81.7 (± 1.2)	3.0 (± 0.5)	33.6 (± 1.3)	11.6 (± 0.7)	138.4 (± 4.0)	17.1 (± 0.4)	8.5 (± 0.1)	34.4 (± 1.2)	28.6 (± 0.7)
3	26	2 (3–6)	102.0 (± 3.0)	8.7 (± 1.1)	42.9 (± 1.3)	15.6 (± 1.1)	183.6 (± 3.8)	18.4 (± 0.2)	8.8 (± 0.2)	32.4 (± 0.8)	25.7 (± 1.2)
4	33	3 (3–6–24)	121.0 (± 1.7)	13.9 (± 2.1)	50.0 (± 0.9)	22.5 (± 1.9)	213.6 (± 2.0)	21.0 (± 0.8)	9.7 (± 0.4)	30.8 (± 1.4)	18.1 (± 0.2)
5	40	4 (3–6–24–48)	135.0 (± 0.0)	17.9 (± 1.0)	56.0 (± 0.5)	27.9 (± 1.3)	235.5 (± 3.0)	26.2 (± 0.4)	10.8 (± 0.2)	27.2 (± 0.4)	16.9 (± 0.6)
6^a^	40	4 (3–6–24–48)	146.7 (± 9.2)	15.6 (± 2.0)	58.7 (± 3.6)	25.9 (± 2.6)	251.9 (± 10.2)	21.7 (± 0.5)	9.4 (± 0.3)	30.7 (± 0.1)	18.3 (± 0.3)

^a^The enzyme blend was fed four times simultaneously with the substrate

^b^The fed-batch enzymatic hydrolysis was initiated at 12% (w/v) solids loading and 7% (w/v) substrate was fed different times

The chemical composition of the residues was analyzed and the results are presented in Table 2. Interestingly, the glucan and xylan contents of the residues after the batch hydrolysis at 12% (w/v) solids loading were reduced significantly (P < 0.05) to 14.4% and 7.8%, respectively, compared to the pretreated corn stover. In contrast, the lignin and ash contents dramatically increased (Table 2, Entry 1). This was primarily attributed to the degradation of the carbohydrate polymers within corn stover. When the feeding times rose from 1 to 4, the glucan and xylan contents of the residues increased gradually, while the lignin and ash contents decreased on a continued basis (Table 2, Entries 2–5), which was accordant with the continued decline of the hydrolytic yields (Fig. 3), implying that stronger products inhibition occurred with the increase in solids loadings.

### Effects of enzyme supplementation modes on fed-batch enzymatic hydrolysis

**Fig. 4:**
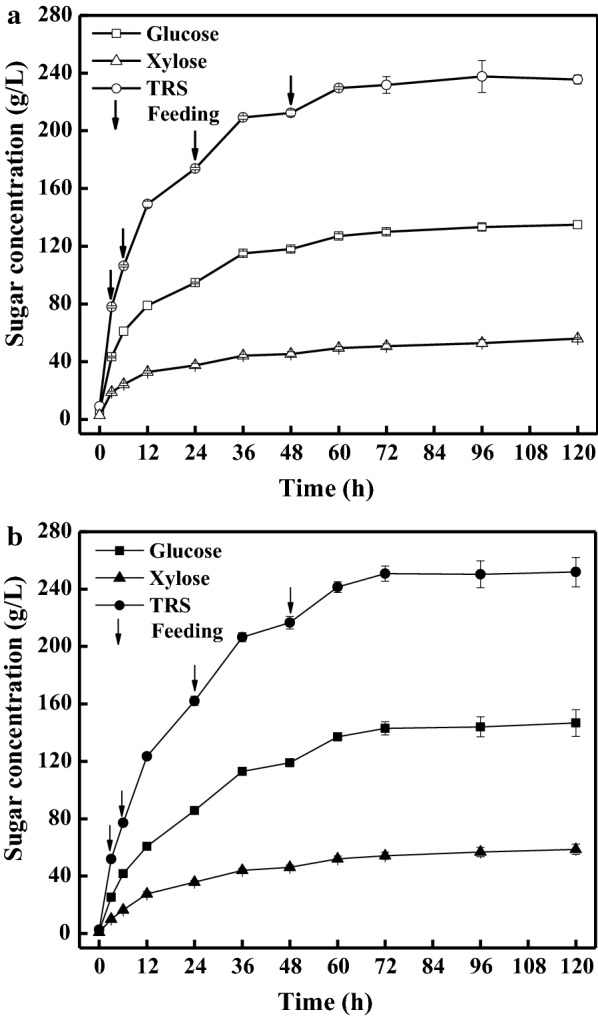
Effects of enzyme supplementation modes on the release of glucose, xylose, and TRS during the fed-batch enzymatic hydrolysis. The fed-batch hydrolysis was initiated at 12% (w/v) solids loading, and 7% fresh substrate was fed four times (3, 6, 24, and 48 h) to reach 40% (w/v) solids loading. The enzyme supplementation modes were as follows: **a** The enzyme blend (based on the final solids loading, 40%, w/v) was added completely at the beginning of the hydrolysis (hollow symbols). **b** The enzyme blend was fed simultaneously with the pretreated corn stover at each time point of feeding to maintain an identical final enzyme dosage (solid symbols)

**Fig. 5:**
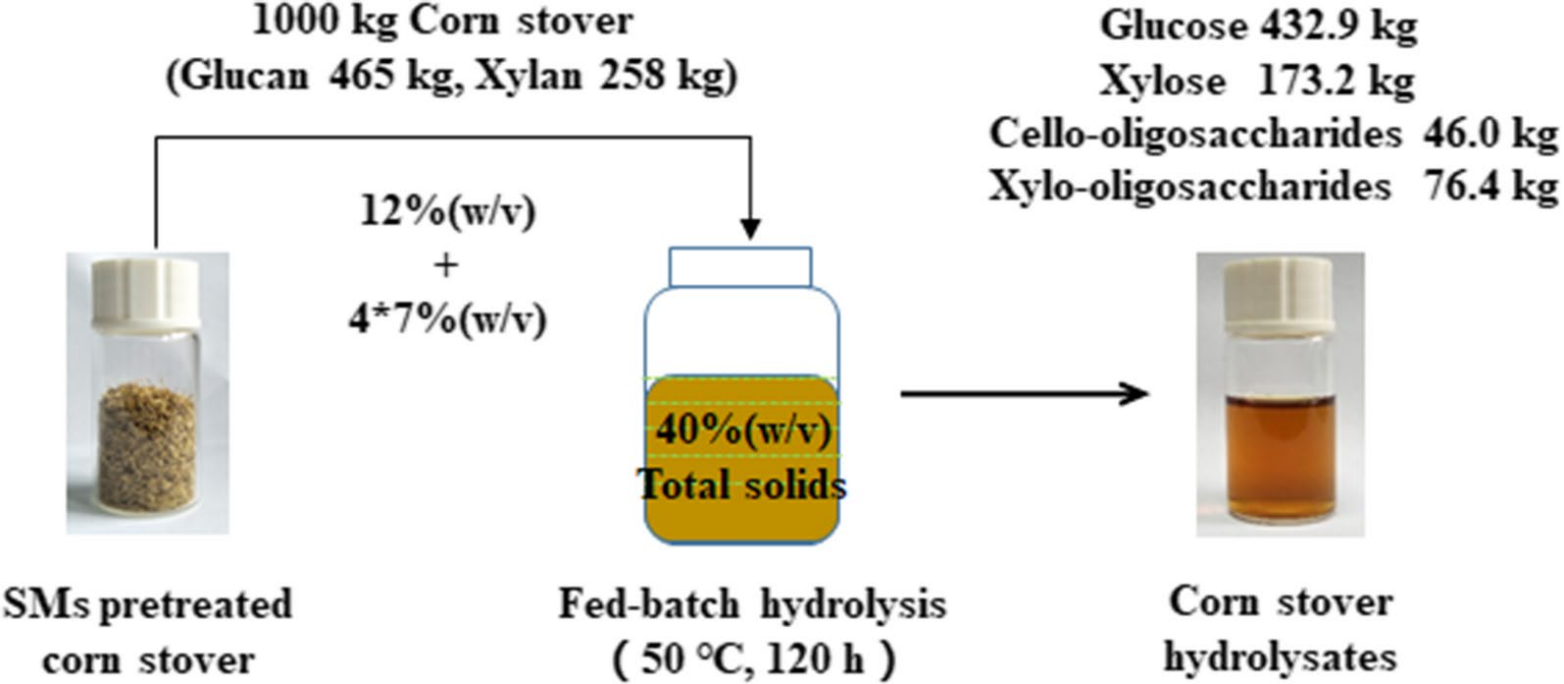
Corn stover to fermentable sugars mass balance analysis. The analysis was based on the fed-batch enzymatic hydrolysis of the SMs-pretreated corn stover. The hydrolysis was initiated at 12% (w/v) solids loading, and 7% substrate was fed four times (3, 6. 24, and 48 h) to reach a final solids loading of 40% (w/v). The enzyme cocktail was fed simultaneously with the substrate at each feeding time point. The hydrolysis was held at 50 °C, pH 4.8, and 200 rpm for 120 h

The effects of enzyme supplementation modes on the hydrolysis yields were investigated and compared during the fed-batch hydrolysis process. The enzyme cocktail was either added completely at the start of the hydrolysis or separately supplemented with the substrate. The fed-batch process was initiated at 12% (w/v) solids loading, and fed four times to reach 40% (w/v) solids loading. As depicted in Fig. 4, more sugars were released when the enzyme was added in a whole within the first 36 h. It was likely more loaded enzyme contributed as a single boost of a higher enzymatic hydrolysis rate. This phenomenon was consistent with the previous reports that cellulase added in a whole facilitated the release of glucose and xylose [16]. However, the rates of sugars release became inferior to those of the fed-batch enzyme supplementation mode after 12 h (Fig. 4), suggesting that the enzymatic activity diminished gradually as the enzymatic hydrolysis proceeded. Interestingly, more sugars were observed when the hydrolysis reaction was extended to 48 h or longer when the required enzyme was added concomitantly with the substrate. It was probably due to a higher enzymatic activity at the later stage of hydrolysis. The maximal concentrations of glucose and xylose were 146.7 g/L and 58.7 g/L, respectively, when the substrate and the enzyme cocktail were both fed separately (Table 2, Entry 6). These data resulted 8.7% and 4.8%, respectively, higher than those with the enzyme added in a whole. The fed-batch addition mode of the enzyme cocktail caused the glucan and xylan contents of the residues to decrease, which was in agreement with the results of the higher sugars concentrations (Table 2, Entry 5 vs Entry 6). Cello-oligosaccharides and xylo-oligosaccharides slightly decreased by 12.8% and 4.8%, while the total conversions of glucan and xylan increased to 89.5% and 88.5%, respectively (Fig. 3). Surprisingly, these conversions were only marginally lower than those from the batch hydrolysis at 12% (w/v) solids loading without significant differences (P > 0.05). According to the mass balance analysis, 432.9 kg glucose, 173.2 kg xylose, 46.0 kg cello-oligosaccharides, and 76.4 kg xylo-oligosaccharides, respectively, could be obtained from 1000 kg of the pretreated corn stover (Fig. 5). These data strongly supported that the enzyme blend added separately during the fed-batch hydrolysis facilitated higher sugars concentrations and yields.

The results of enzymatic hydrolysis on various lignocellulosic materials at high solids loadings under different hydrolysis modes obtained in previous studies and in this project are summarized in Table 3. Some pretreatments remove lignin and hemicellulose while retaining and concentrating cellulose in the regenerated solids, as an approach favorable to high glucose concentrations with the fed-batch enzymatic hydrolysis (Table 3, Entries 1 and 2). However, the high glucose concentration exerts a substantial inhibitory effect on cellulase, which could affect the hydrolysis efficiency of cellulose [28]. For example, the total conversion was merely 60.0%, when the fed-batch enzymatic hydrolysis of corn stover was performed at 30% (w/v) solids loading (Table 3, Entry 2). Xylose and oligosaccharides have been directly used for the production of biofuels [31, 32]. Some microorganisms are capable to metabolize a broad spectrum of substrates and can grow on a medium containing glucose and other sugars such as xylose, cello-oligosaccharides, and xylo-oligosaccharides. Here, the total conversions of glucan and xylan reached approximately 89% taking into account the oligosaccharides released. The SMs pretreatment can detain a majority of the sugar polymers including cellulose and hemicelluloses [33]. Here, the oligosaccharides released from corn stover were up to 41.5 g/L, which contributed to the high conversions of glucan and xylan (Table 3, Entry 5). The total sugar conversions were slightly higher than the monomeric sugar yields achieved by the batch hydrolysis with the assistance of mechanical stirring (Table 3, Entry 3). Overall, the fed-batch enzymatic hydrolysis strategy was promising for hydrolyzing alkaline organosolv-pretreated corn stover into fermentable sugars with high concentrations and yields.

**Table 3:** The results of enzymatic hydrolysis on various lignocellulosic materials at high solids loadings using different hydrolysis modes

Entry	Hydrolysis mode	Lignocellulosic materials	Pretreatment methods	Solids loading	Hydrolysis time (h)	Sugar concentration (g/L)					Conversion rate	References
						Glucose	Xylose	Cello-oligosaccharides	Xylo-oligosaccharides	TRS		
1	Fed-batch	Corncob	Irradiation pretreatment	40% (w/v)	120	235	16	_	_	251	_	[20]
2	Fed-batch	Corn stover	Steam explosion and alkaline hydrogen-peroxide pretreatment	30% (w/v)	144	175	22	20		220	Total conversion rate of 60%	[28]
3	Batch with mechanical stirring	Corn stover	NaOH pretreatment and mechanical refining	28% (w/w), equals to 38.9% (w/v)	120	135.2	99.4	7.3	_	270	Monomeric sugar yield of 87%	[12]
4	Fed-batch	Sugarcane bagasse	Alkali pretreatment	33% (w/v)	120	129.5	56.0	9.4	_	200	Glucan conversion of 59.9%	[16]
5	Fed-batch	Corn stover	SMs pretreatment	40% (w/v)	120	146.7	58.7	15.6	25.9	251.8	Glucan and xylan conversions of 89.5% and 88.5%, respectively	This work

### Conversion of the monosaccharides and oligosaccharides within the hydrolysates into microbial lipids using a fed-batch fermentation mode

**Fig. 6:**
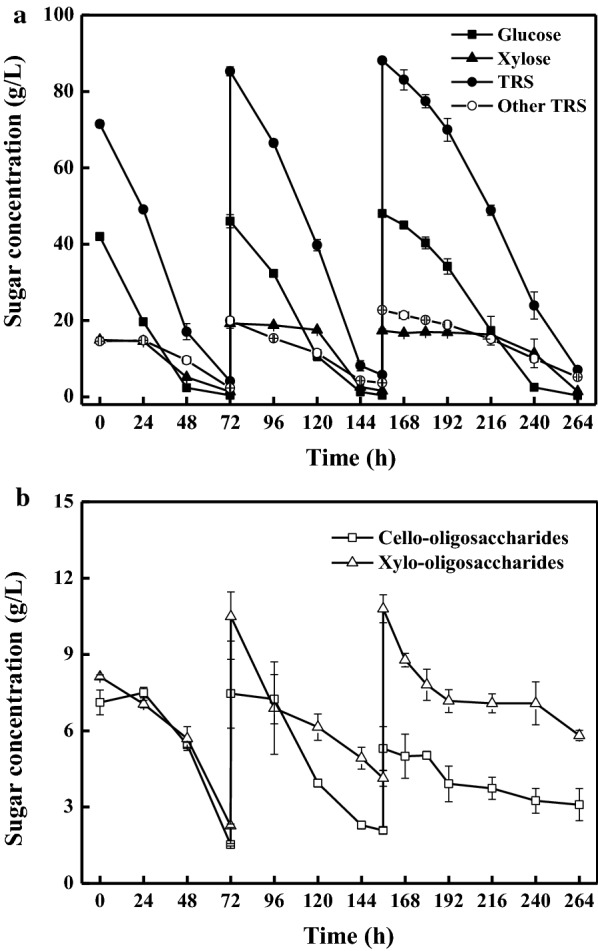
Time course of sugars evolution during the fed-batch culture by *C. oleaginosum* on corn stover enzymatic hydrolysates. The culture was held at 30 °C, 200 rpm for 264 h. Corn stover hydrolysates containing 244.5 g/L TRS were fed at 72 h and 156 h, respectively

Fed-batch fermentation is a promising culture mode for the industrial-scale lipid production [36]. In this study, cell growth and lipid accumulation were severely repressed when the TRS concentration of the hydrolysate was 150 g/L during batch fermentation. More severely inhibitory effect occurred at even higher sugar concentration (Additional file 3). Thus, a fed-batch culture was investigated to assess the potential of sugars consumption and lipid production by *C. oleaginosum*. The fed-batch culture was held at 30 °C, 200 rpm for 264 h. Sugar evolution profiles of the fed-batch culture are shown in Fig. 6. When glucose concentration was higher than 20 g/L, glucose was assimilated firstly, whereas xylose was assimilated at quite a slow rate without significant consumption (P < 0.05) (Fig. 6a). It was suggested that *C. oleaginosum* exhibited a diauxic growth with a strong preference over glucose. A similar phenomenon was observed when glucose and xylose coexisted, suggesting the occurrence of glucose repression [40]. As shown in Fig. 6a, the other TRS (subtraction of glucose and xylose from the TRS) were consumed slowly. It was worth mentioning that 87.8% of them were assimilated at the end of the fed-batch culture. The main components of these TRS were arabinose, galactose, mannose, soluble oligosaccharides, etc. Interestingly, both cello-oligosaccharides and xylo-oligosaccharides were assimilated by *C. oleaginosum* (Fig. 6b). The overall consumption of these two types of sugars was 74.1% and 68.3%, respectively (Table 4). Only 3.1 g/L cello-oligosaccharides and 5.8 g/L xylo-oligosaccharides were not consumed, likely being soluble oligosaccharides with higher degree of polymerization [31]. The assimilation of oligosaccharides gradually decreased along with the fermentation, mainly due to the decline of the cell activity at the later stage of fermentation. The highest values of biomass, lipid concentration, and lipid content reached 50.7 g/L, 31.3 g/L, and 61.7% (w/w), respectively. The overall lipid yield was 0.18 g/g TRS, corresponding to a carbon yield of 34.8%. The theoretical lipid yields on glucose and xylose are 0.33 g/g and 0.34 g/g [41], corresponding to theoretical carbon yields of 63.8% and 65.7%, respectively. However, the experimental data were rarely higher than 0.22 g/g glucose, corresponding to a carbon yield of 42.5% [42]. Recently, Fei and coworkers have reported a fed-batch culture using an automated online sugar control feeding mode, which obtained a very high lipid yield of 0.29 g/g [43]. Thus, the lipid yield has some space to be further optimized in bioreactor by altering the culture conditions and the nutrients compositions of the media, which may exert significant effects on carbon-flux distribution between cell growth and lipid biosynthesis.

**Table 4:** Fed-batch culture results of *C. curvatus* on the corn stover enzymatic hydrolysates

Entry	Time (h)	DCW (g/L)	Lipid concentration^a^ (g/L)	Lipid content^b^ (%, w/w)	Lipid yield^c^ (g/g)	Cello-oligosaccharides consumption (%, w/w)	Xylo-oligosaccharides consumption (%, w/w)
1	72	30.8 ± 0.3	9.4 ± 0.1	30.4 ± 0.5	0.14 ± 0.00	78.6 ± 1.8	72.1 ± 1.1
2	156	43.1 ± 0.2	21.7 ± 0.1	50.3 ± 0.1	0.17 ± 0.00	81.7 ± 2.1	71.7 ± 2.9
3	264	50.7 ± 1.0	31.3 ± 0.6	61.7 ± 0.2	0.18 ± 0.00	74.1 ± 5.1	68.2 ± 1.1

^a^Lipid concentration is defined as gram lipid per liter culture broth

^b^Lipid content is calculated as gram lipid per g DCW

^c^Lipid yield is calculated as gram lipid produced per gram carbon sources consumed

Lignocellulose-derived oligosaccharides cannot be metabolized by some microorganisms and will be discharged along with fermentation wastewater, which means the loss of sugars. Xue and coworkers have stated that the release of oligosaccharides indicates a yield loss as they are recalcitrant for a majority of industrial ethanol-producing microorganisms [22]. High proportion of oligosaccharides is released when the enzymatic hydrolysis of lignocelluloses is conducted at high solids loadings. For example, one-third of the total output sugars were oligomers or polymers when the enzymatic hydrolysis was conducted at 17.6% (w/w) solids loading although the commercial enzymes mixtures were loaded [21]. In this study, 16.8% of the total soluble sugars within the hydrolysate were oligosaccharides when the fed-batch enzymatic hydrolysis was conducted at the final solids loading of 40% (w/v), although the enzyme blend contained very high cellobiase activity (253.0 CBU/gds). The accumulation of oligosaccharides is expected to be lower when the enzyme cocktail is optimized. However, it is difficult and uneconomical to completely avoid the release of oligosaccharides during the enzymatic hydrolysis. Development of oligosaccharides assimilation strains will be particularly advantageous as a sizeable portion of sugars in the hydrolysate are oligomers. Here, the oligosaccharides could be co-fermented with the monosaccharides (glucose and xylose), evidencing that the sugar loss can be avoided. In addition, extra enzymes were not deemed necessary during the hydrolysis process, which is conducive to the techno-economics of the enzymatic hydrolysis technology. High sugar concentrations and yields of fermentable sugars were reached using the fed-batch hydrolysis with a high enzyme dosage (15 mg protein/g glucan). The high enzyme cost severely impedes the commercial success. Nguyen and coworkers have reported that simultaneous saccharification and fermentation (SSF) of corn stover at high solids loadings achieved high ethanol titers with significantly reduced enzyme dosages [44, 45]. The integration of the fed-batch enzymatic hydrolysis and lipid fermentation has the opportunity to reach high lipid yield with significantly lower enzyme loadings.

Lipid samples produced by *C. oleaginosum* using the fed-batch culture were transmethylated, and the resulting FAMEs were analyzed by GC. The major composition of fatty acids was long-chain fatty acids with 16 and 18 carbon atoms including myristic acid (0.7 ± 0.0%), palmitic acid (27.5 ± 0.4%), stearic acid (12.0 ± 0.3%), oleic acid (49.0 ± 0.2%), and linoleic acid (9.6 ± 0.2%). Specifically, oleic acid was the most abundant one, followed by palmitic, stearic, and linoleic acid, and these four fatty acids accounted for over 98% of the total fatty acids. The lipid samples presented a fatty acid profile similar to those of vegetable oils, demonstrating that the microbial lipid could be explored as a potential feedstock for biodiesel production [46].

## Conclusion

The fed-batch enzymatic hydrolysis of alkaline organosolv-pretreated corn stover was promising for obtaining high concentrations and yields of fermentable sugars. The total conversions of glucan and xylan reached 89.5% and 88.5%, respectively, at a high solids loading of 40% (w/v), when the oligosaccharides released were taken into account. High lipid content and yield were realized under a fed-batch culture mode. Oligosaccharides were co-assimilated with monosaccharides by *C. oleaginosum*, which played a crucial role in minimizing the loss of oligosaccharides and improving the lipid production. This work provides valuable information facilitating the design of a more cost-effective lignocellulose-to-lipid route.

## Methods

### Raw materials, enzymes, and chemicals

Corn stover used in this study was purchased from Zhengzhou (Henan, China). It was chopped by a high-speed shredder (HC-3000, Wuyi Haina Electric Appliance Co., Ltd., Jinhua, China) before sieving through 1-mm-sized screen. Then, the samples were washed for three times with water to remove the adhering dirt, stones, and metals, oven-dried at 105 °C until the weight was constant, and stored in a desiccator for long-term storage. The raw corn stover contained 35.9% glucan, 21.7% xylan, 22.1% total lignin, and 2.3% ash (Table 1, Entry 1). The other materials were proteins, non-structural sugars, organic acid, inorganics, crude fat, waxes, coloring material, etc. Specifically, there were 8.7% water extractives and 4.5% ethanol extractives, respectively, in the raw corn stover. Cellic^®^ CTec2, a commercial enzyme blend containing aggressive cellulases, high level of β-glucosidase, and hemicellulase [47], was purchased from Novozymes (Tianjin, China). The total protein concentration of the enzyme blend was determined to be 116.2 mg/mL. The filter paper activity and β-glucosidase activity were 247.1 FPU/mL (filter paper units per milliliter of enzyme solution) and 4167 CBU/mL (cellobiase units per milliliter of enzyme solution), respectively, as assayed by the published procedures [48, 49]. The chemical reagents used in this study were of analytical grade and purchased locally.

### Alkaline organosolv pretreatment procedure

The SMs pretreatment of corn stover at high solids loading was conducted according to our previous published method with some modifications [33]. Specifically, NaOH (4.0 g) and methanol (80 mL) of analytical grades were mixed in a 500-mL red cap glass bottle (Duran, Schott, Germany) to obtain a homogeneous solution. 40 g of the raw corn stover was added into the bottle and mixed thoroughly with the solution by stirring with glass rods. Then, the bottle was sealed and immersed in a water bath (80 °C) for 1 h. When the pretreatment was complete, the slurry was cooled below 50 °C by water instantly, filtrated through a Buchner funnel, and washed for three times with 400 mL of water to a weak alkalinity (pH 9.65). Finally, the pretreated corn stover was oven-dried at 105 °C to constant weight and sieved using a 1-mm-sized screen prior to use.

### Batch enzymatic hydrolysis

The SMs-pretreated corn stover was resuspended in the citrate buffer (50 mM, pH 4.8) in a 100-mL red cap bottle (Duran, Schott, Germany) to achieve solids loadings ranging from 2.5 to 40% (w/v). The total liquid phase including the buffer and enzyme solution was 20 mL. Unless otherwise specified, solids loading (w/v) refers to the ratio of the mass (g) of corn stover to the volume (mL) of the aqueous phase added to prepare the suspension. The enzyme blend was loaded at 15 FPU/gds (gram of initial dry substrate), corresponding to 7 mg protein/gds or 15 mg protein/g glucan. Batch enzymatic hydrolysis was performed in triplicates at 50 °C in a water bath oscillator (SHZ-88, Jiangsu JinYi Instrument Technology Co., Ltd., Changzhou, China) constantly agitated at 200 rpm. Unless otherwise stated, 0.3% (w/v) sodium azide was supplemented to prevent bacterial contamination. At different time points of hydrolysis, the samples were drawn and centrifuged at 10,000*g* for 3 min and the resulting supernatant was used for sugars analysis. A known volume of deionized water was added to the hydrolytic slurries before centrifugation since there was no visible liquid phase when the batch enzymatic hydrolysis was directly performed at high solids loading of 33% or 40%. The true sugar concentration was calculated taking into account the dilution.

### Fed-batch enzymatic hydrolysis

The fed-batch enzymatic hydrolysis of corn stover pretreated by the SMs was initiated at 12% (w/v) solids loading in a 100-mL red cap bottle (Duran, Schott, Germany). The total liquid phase including the buffer and enzyme solution was 20 mL. The enzyme blend was loaded at 15 mg protein/g glucan. Unless otherwise specified, all the required enzyme based on the final substrate loadings was added in a whole at the beginning of the hydrolysis. The hydrolysis was performed in triplicates at 50 °C and pH 4.8 in a water bath oscillator of 200 rpm. In order to identify the time point of feeding, the pretreated corn stover (7%, w/v) was separately fed at the time point of 3, 6, 12, 24, or 36 h, respectively, to achieve the final solids loading of 19% (w/v).

For fed-batch enzymatic hydrolysis with higher solids loadings, the hydrolysis was initiated at 12% (w/v) solids loading and the pretreated corn stover (7%, w/v) was fed once (at 3 h), twice (at 3 and 6 h), three times (at 3, 6, and 24 h), or four times (at 3, 6, 24, and 48 h) to reach the solids loadings of 19%, 26%, 33%, or 40% (w/v), respectively. For the solids loading of 40%, the required enzyme was supplemented in two modes, either added in a whole at the beginning of the hydrolysis or fed simultaneously with the substrate at the time points of 0, 3, 6, 24, and 48 h to maintain an identical final enzyme dosage.

### Microorganism and pre-culture preparation

*Cutaneotrichosporon oleaginosum* (formerly *Cryptococcus curvatus*) ATCC 20509 was sourced from the American Type Culture Collection (ATCC), stored at 4 °C and propagated every 4 weeks on yeast peptone dextrose (YPD) agar slants (yeast extract 10 g/L, peptone 10 g/L, glucose 20 g/L, agar 15 g/L, pH 6.0). Pre-cultures were grown in 50 mL YPD liquid medium at 30 °C for 24 h unless otherwise specified.

### Fed-batch culture

The fed-batch cultures were conducted in 250-mL unbaffled Erlenmeyer flasks. The cultures were inoculated with the addition of 2.5 mL of pre-cultures to 22.5 mL appropriately diluted hydrolysates containing 70 g/L TRS, 10 g/L yeast extract, and 10 g/L peptone. The initial pH was set at 6.0. The cultures were incubated at 30 °C and 200 rpm in a thermostatic culture oscillator (ZWY-2102, Shanghai Zhicheng Analytical Instrument Manufacturing Co., Ltd., Shanghai, China) for 264 h. Sterile liquid hydrolysates containing 244.5 g/L TRS were then fed twice when residual sugar was below 10 g/L. The pH of the culture was adjusted to the initial value at 12-h intervals. To avoid bacterial contamination, the pH adjustment was performed in a clean bench using a pH meter (PB-10, Sartorius, Germany). The pH electrode was rinsed repeatedly with sterile water prior to use.

All the enzymatic hydrolysis and fermentation experiments were performed in triplicates. The data were presented as mean value ± standard deviation.

### Analytical method

Glucose was measured using a biosensor analyzer (SBA-40E, Shandong Academy of Sciences, Jinan, China). Xylose was determined by a Megazyme D-xylose assay kit (K-XYLOSE, Megazyme, Ireland). The concentration of TRS was determined by the dinitrosalicylate (DNS) method described by Miller [50]. Other TRS concentration was obtained by subtracting glucose and xylose from the TRS. The concentration of total protein was measured according to a Coomassie Brilliant Blue method [51].

The concentrations of cello-oligosaccharides and xylo-oligosaccharides were measured according to a standardized Laboratory Analytical Procedure (LAP) described by National Renewable Energy Laboratory (NREL) with some modifications [22, 52]. Specifically, 2 mL of appropriately diluted hydrolysates and the glucose/xylose recovery standards were added into 10-mL screw-cap glass vials. 69.7 μL of 72% (w/w) sulfuric acid was then added into the bottle to obtain a final acid concentration of 4%. The bottle was then autoclaved at 121 °C for 1 h. The samples were slowly cooled to room temperature, neutralized with calcium carbonate powder to pH between 5 and 6, and filtered through a 0.2-μm filter before sugars analysis. The sugar recovery standards were used to correct for losses due to destruction of sugars during dilute acid hydrolysis, which were calculated as the percentage of the remaining sugars after the hydrolysis to the initial values. The concentrations of cello-oligosaccharides ($c_{{ [ {\text{cello - oligo]}}}}$, g/L) and xylo-oligosaccharides ($c_{{ [ {\text{xylo - oligo]}}}}$, g/L) were calculated according to the following equations:

1 $$c_{{ [ {\text{cello-oligo]}}}} \left( {\text{g/L}} \right) = \frac{{[c'_{{ [ {\text{glu]}}}} {\text{ (g/L)}} \times v'{\text{ (L)/}}R_{{ [ {\text{glu]}}}} { - }c_{{ [ {\text{glu]}}}} {\text{ (g/L)}} \times v{\text{ (L)]}}}}{{v{\text{ (L)}}}} \times 0.9$$

2 $$c_{{ [ {\text{xylo-oligo]}}}} \left( {\text{g/L}} \right) = \frac{{[c'_{{ [ {\text{xyl]}}}} {\text{ (g/L)}} \times v'{\text{ (L)/}}R_{{ [ {\text{xyl]}}}} { - }c_{{ [ {\text{xyl]}}}} {\text{ (g/L)}} \times v{\text{ (L)]}}}}{{v{\text{ (L)}}}} \times 0.88$$

$c_{{ [ {\text{glu]}}}}$ and $c_{{ [ {\text{xyl]}}}}$ are the initial concentrations (g/L) of glucose and xylose in the liquid hydrolysates, respectively. $c'_{{ [ {\text{glu]}}}}$ and $c'_{{ [ {\text{xyl]}}}}$ are the concentrations (g/L) of glucose and xylose after the acid hydrolysis, respectively. $v$ and $v'$ are the volumes (L) of the hydrolysates before and after the acid hydrolysis, respectively. $R_{{ [ {\text{glu]}}}}$ and $R_{{ [ {\text{xyl]}}}}$ are the recovery of glucose and xylose standards after the acid hydrolysis divided by the initial values. The content of the polymeric sugars was calculated from the corresponding monosaccharides, using an anhydro correction of 0.90 (162/180) for glucose and 0.88 (132/150) for xylose.

A two-step extraction process with water followed by ethanol was performed using a published Laboratory Analytical Procedure documented by NREL [53]. Ash content was determined by a dry oxidation method using the NREL procedure [54]. A two-step acid hydrolysis method was applied for the determination of glucan, xylan, and total lignin (the sum of acid insoluble lignin and acid soluble lignin) content of corn stover according to a published procedure of NREL [55].

Glucose yield ($Y_{\text{glu}}$, %), cello-oligosaccharides yield ($Y_{\text{cello - oligo}}$, %), xylose yield ($Y_{\text{xyl}}$, %), xylo-oligosaccharides yield ($Y_{\text{xylo - oligo}}$, %), total conversion of glucan ($\alpha_{\text{glucan}}$, %), and total conversion of xylan ($\alpha_{\text{xylan}}$, %) were calculated according to the following equations:

3 $$Y_{\text{glu}} \left( {\text{\% }} \right) = \frac{{[c_{{ [ {\text{glu]}}}} {\text{ (g/L)}} \times v{\text{ (L) }} - m_{\text{glu}}^{\text{enzyme}} ( {\text{g)]}} \times 0. 9 0}}{{m_{\text{pretreated corn stover}} ( {\text{g)}} \times F_{\text{glucan}} ( {\text{\% )}}}} \times 100$$

4 $$Y_{\text{cello-oligo}} \left( {\text{\% }} \right) = \frac{{[c_{{ [ {\text{cello-oligo]}}}} {\text{ (g/L)}} \times v{\text{ (L) }} - m_{\text{glu-oligo}}^{\text{enzyme}} ( {\text{g)]}}}}{{m_{\text{pretreated corn stover}} ( {\text{g)}} \times F_{\text{glucan}} ( {\text{\% )}}}} \times 100$$

5 $$Y_{\text{xyl}} \left( {\text{\% }} \right) = \frac{{c_{{ [ {\text{xyl]}}}} {\text{ (g/L)}} \times v{\text{ (L)}} \times 0. 8 8}}{{m_{\text{pretreated corn stover}} ( {\text{g)}} \times F_{\text{xylan}} ( {\text{\% )}}}} \times 100$$

6 $$Y_{\text{xylo-oligo}} \left( {\text{\% }} \right) = \frac{{c_{{ [ {\text{xylo - oligo]}}}} {\text{ (g/L)}} \times v{\text{ (L) }}}}{{m_{\text{pretreated corn stover}} ( {\text{g)}} \times F_{\text{xylan}} ( {\text{\% )}}}} \times 100$$

7 $$\alpha_{\text{glucan}} \left( {\text{\% }} \right) = Y_{\text{glu}} \left( {\text{\% }} \right) + Y_{\text{cello-oligo}} \left( {\text{\% }} \right)$$

8 $$\alpha_{\text{xylan}} \left( {\text{\% }} \right) = Y_{\text{xyl}} \left( {\text{\% }} \right) + Y_{\text{xylo-oligo}} \left( {\text{\% }} \right)$$

High concentrations of glucose and other sugars are present in Cellic^®^ CTec2 solution to facilitate the structural stability and the long-term storage of the enzymes. Glucose and oligosaccharides containing glucose unit (glu-oligo) of 276.7 g/L and 72.4 g/L, respectively, were observed in the enzyme blend. Thus, $m_{\text{glu}}^{\text{enzyme}}$ and $m_{\text{glu - oligo}}^{\text{enzyme}}$ are the masses (in grams) of glucose and glu-oligo existing in the enzyme supplied. $m_{\text{pretreated corn stover}}$ is the mass (in grams) of the pretreated corn stover. $F_{\text{glucan}}$ and $F_{\text{xylan}}$ represent the mass ratios (%, w/w) of glucan and xylan to the pretreated corn stover.

The density and volume of the liquid hydrolysates continuously increased, with sugars and other substances constantly dissolving into the corn stover hydrolysates. The sugars yields would be underestimated when they were calculated by assuming the volume of the liquid phase not changed. The higher the solids loadings applied, the bigger the underestimation. Here, the sugars yields were corrected by multiplying the exact volumes. The density ($\rho$, g/L) and volume ($v$, L) were calculated according to Eqs. (9) and (10), respectively, based on the published methods with minor modifications [56–58].

9 $$\rho {\text{ (g/L)}} = { 0} . 3 7 7\times [c_{{ [ {\text{glu]}}}} ( {\text{g/L) + }}c_{{ [ {\text{cello-oligo]}}}} ( {\text{g/L) + }}c_{{ [ {\text{xyl]}}}} ( {\text{g/L) + }}c_{{ [ {\text{xyl-oligo]}}}} ( {\text{g/L)] + }}\rho_{\text{w}}$$

10 $$v{\text{ (L) }} = \frac{{[m_{\text{liquid}} {\text{ (g)}} + m_{\text{regenerated corn stover}} {\text{ (g)}} - m_{\text{residues}} {\text{ (g)}}]}}{{\rho {\text{ (g/L)}}}}$$

$\rho$ and $\rho_{\text{w}}$ are the densities (g/L) of the liquid hydrolysates and water, respectively. $m_{\text{liquid}}$ and $m_{\text{residues}}$ are the masses (in grams) of the liquid phase and the insoluble solids after the enzymatic hydrolysis, respectively.

Biomass, expressed as dry cell weight (DCW), was determined by a gravimetric method [40]. Lipids were extracted twice from the dry cells using a mixture of chloroform and methanol (1:1, v/v) and measured by a gravimetric method in accordance with a published procedure [40]. Lipid concentration was presented as gram lipid per liter of culture. Lipid content was expressed as gram lipid per gram DCW. Lipid yield was calculated as gram lipid accumulated per gram TRS consumed.

To analyze the fatty acid compositions, the microbial lipid samples were transesterified with methanol in the presence of KOH according to a published procedure [59]. The resulting fatty acid methyl esters (FAMEs) were determined using a gas chromatograph (GC-2010 Plus, Shimadzu, Japan) following a standard procedure [60].

### Statistical analysis

The significant differences were identified by conducting an analysis of variance (ANOVA). Tukey’s post hoc test was performed to determine the significant differences among the data as obtained from different enzymatic hydrolysis experiments. A p value lower than 0.05 was treated as statistically significant.

## Supplementary information

**Additional file 1.** Time course of sugar evolution during the batch enzymatic hydrolysis at solids loadings ranging from 8 to 14% (w/v).

**Additional file 2.** Sugar evolution profiles of fed-batch enzymatic hydrolysis with different feeding times.

**Additional file 3.** Effects of initial sugar concentrations of the hydrolysate on the cell growth and lipid production by *C. oleaginosum*.

## Data Availability

All appropriate data for this study have been included in the manuscript.

## Abbreviations

SMs: the sodium hydroxide–methanol solution
FPU: filter paper unit
CBU: cellobiase unit
TRS: total reducing sugars
DNS: dinitrosalicylate
NREL: the National Renewable Energy Laboratory
DCW: dry cell weight
FAMEs: fatty acid methyl esters
ANOVA: an analysis of variance
AFEX: ammonia fiber explosion
SSF: simultaneous saccharification and fermentation
