# Supplementary material for: Fed-batch enzymatic hydrolysis of alkaline organosolv-pretreated corn stover facilitating high concentrations and yields of fermentable sugars for microbial lipid production
Source: Biotechnol Biofuels. 2020 Jan 22;13:13. doi: 10.1186/s13068-019-1639-9 (PMC6977323; doi:10.1186/s13068-019-1639-9)
Supplement: Supplementary file 1 — Additional file 1. Time course of sugar evolution during the batch enzymatic hydrolysis at solids loadings ranging from 8 to 14% (w/v). [file 13068_2019_1639_MOESM1_ESM.doc]

**Fig. S1** **Time course of sugar evolution during the batch enzymatic hydrolysis of corn stover at solids loadings ranging from 8% to 14% (w/v).** Cellic® CTec2 was loaded at 15 mg protein/g glucan. The pretreated corn stover was hydrolyzed at 50°C, pH 4.8 for 12 h. As shown in Fig. S1, glucose concentration exhibited a highly significant linear relationship (y = 1.63x + 5.30, R^2^ = 0.9998) with solids loadings ranging from 8% to 12%, when the enzymatic hydrolysis was held for 3 h. However, the percentage increment was reduced significantly (P<0.05) when the solids loading was 14%. In addition, the slurries were quickly liquefied within a space of 3 h when the solids loadings ranged from 8% to 12%, whereas the time of liquefaction became retarded to 6 h at the solids loading of 14%. Thus, the initial solids loading was set to 12% in view of the initial hydrolytic efficiency.
