# Supplementary material for: Fed-batch enzymatic hydrolysis of alkaline organosolv-pretreated corn stover facilitating high concentrations and yields of fermentable sugars for microbial lipid production
Source: Biotechnol Biofuels. 2020 Jan 22;13:13. doi: 10.1186/s13068-019-1639-9 (PMC6977323; doi:10.1186/s13068-019-1639-9)
Supplement: Supplementary file 2 — Additional file 2. Sugar evolution profiles of fed-batch enzymatic hydrolysis with different feeding times. [file 13068_2019_1639_MOESM2_ESM.doc]

**Fig. S2.** S**ugar evolution profiles of fed-batch enzymatic hydrolysis with different feeding times.** The batch enzymatic hydrolysis was conducted at 12% (w/v) solids loading (A). The fed-batch enzymatic hydrolysis was initiated at 12% (w/v) solids loading, and 7% (w/v) regenerated corn stover was fed once (B), twice (C), three times (D), and four times (E) to get final solids loadings of 19%, 26%, 33%, and 40%, respectively. Cellic® CTec2 (based on the final solids loading) was loaded at 15 mg protein/g glucan and added completely at the beginning of the hydrolysis. The hydrolysis was held at 50 °C, pH 4.8 and 200 rpm for 120 h.
