# Supplementary material for: Fed-batch enzymatic hydrolysis of alkaline organosolv-pretreated corn stover facilitating high concentrations and yields of fermentable sugars for microbial lipid production
Source: Biotechnol Biofuels. 2020 Jan 22;13:13. doi: 10.1186/s13068-019-1639-9 (PMC6977323; doi:10.1186/s13068-019-1639-9)
Supplement: Supplementary file 3 — Additional file 3. Effects of initial sugar concentrations of the hydrolysate on the cell growth and lipid production by C. oleaginosum. [file 13068_2019_1639_MOESM3_ESM.docx]

**Fig. S3 Effects of initial sugar concentrations of the hydrolysate on the cell growth and lipid production by *C. oleaginosum*.** The batch cultures were incubated at 30°C and 200 rpm in a thermostatic culture oscillator. For (A), the optical density of the culture was determined at 600 nm (OD_600nm_) with an appropriate dilution using a UV/vis spectrophotometer (UV-1200, MAPADA, China). For (B), biomass and lipid concentration were analyzed when the culture was terminated at 120 h.
